# Gender differences among discrimination & stigma experienced by depressive patients in Pakistan

**DOI:** 10.12669/pjms.316.8454

**Published:** 2015

**Authors:** Nashi Khan, Rukhsana Kausar, Adeela Khalid, Anum Farooq

**Affiliations:** 1 Nashi Khan, PhD.(PK), Post Doc. (UK). Assistant Professor, Centre for Clinical Psychology, University of the Punjab, Lahore, Pakistan; 2 Rukhsana Kausar, PhD. (UK), Post Doc. (UK). Director, Centre for Clinical Psychology, University of the Punjab, Lahore, Pakistan; 3 Adeela Khalid, PhD Scholar (PU, Lahore). Lecturer, Centre for Clinical Psychology, University of the Punjab, Lahore, Pakistan; 4 Anum Farooq, MS Student (PU, Lahore). Centre for Clinical Psychology, University of the Punjab, Lahore, Pakistan

**Keywords:** Discrimination, Stigma, Mental Illness, Depression, Psychological Treatment

## Abstract

**Objective:**

This study aims to examine Gender Difference in the level of Discrimination and Stigma experienced by people diagnosed with Major Depressive Disorder in Pakistan. It was hypothesized that Women diagnosed with Depression are likely to be experiencing more Discrimination and Internalized Stigma in comparison to Men.

**Methods:**

This is a Cross Sectional Study. Thirty eight patients diagnosed with Major Depressive Disorder recruited from different Government Sector Hospitals of Lahore; were approached after obtaining informed consent. Discrimination and Stigma were measured through Discrimination and Stigma Scale and Internalized Stigma of Mental Illness Inventory respectively.

**Results:**

Both Men and Women experience considerably high level of associated Stigma and Discrimination due to their Mental Illness. However, Women in comparison to Men experience significantly greater level of Internalized Stigma especially in domains of Discrimination Experience and Social Withdrawal.

**Conclusion:**

The findings of this study highlight the fact that people with Depression can be more benefited with psychological treatment if dealing with Stigma and Discrimination is also addressed in Intervention Plans.

## INTRODUCTION

Depression signifies a chief public health dilemma that is described by feeling low, reduced attention and concentration, loss of sense of control and a personal experience of great suffering. Prevalence studies give very elevated figures of Depression worldwide.^1^ It is third foremost contributor to the overall burden of disease.^2^ Stigma associated with Mental Illness has been studied in many researches around the globe. In the West associated Stigma with Mental Illness is well acknowledged now. However in Developing Countries, the Discrimination and Stigma associated with mental illness is still uncovered to a greater extent.^3^

Perceived Stigma is the conviction that the public have negative attitudes towards individuals with mental health issues and the apprehension that others will discriminate them, while Experienced Stigma identifies incidents of unjust treatment due to Mental Health Problem.^4^

There is an evidence to suggest that people from different social classes and professions have negative ideas about psychological patients and disorders.^5^ Loss of comfort and personal wellbeing are not caused completely by the etiologies, in-capabilities, and anxiety induced by their psychological disorder. Stigma and Discrimination associated with illness also hamper Quality of Life, Self Esteem and Personal Sense of Wellbeing. People with psychological problems embrace high level of Stigma in comparison to physical illnesses.^6^

Perceived Stigma is linked with reduced life quality,^7^ poor self-esteem^8^, decreased occupational functioning and increased hindrances for social and leisure activities.^7^,^9^ It is also related with greater severity of Depression Level^9^,^10^ and reduced Treatment Adherence.^9^ A research was conducted on attitudes of teachers and students in a University of Lahore, Pakistan towards mental illness revealed that most of the participants had negative attitudes towards mental illnesses specifically towards Schizophrenia, Depression, and Substance Abuse.^11^ Even 50 % of the medical students and doctors held negative attitudes towards Mental Illness.

In Pakistan, a country of 175 million people there are only 800 Mental Health Practitioners and four Mental Hospitals. Patients from rural areas have no access to psychological services. In addition in Pakistan, people avoid seeking psychological help due to the Social Stigma faced by a person who visits a Psychologist or a Psychiatrist.^12^

In Developing Countries, the Culture and Gender plays a complex role in shaping the Stigma associated with Mental Illnesses. In addition in developing countries, mental illnesses are also viewed as the impact of supernatural forces. Such faulty explanations also complicate the issue and become the roots of Stigmatization.^11^ Gender Differences and associated Discrimination with Mental Illnesses has caught very little attention of the researchers.^13^ This situation raises many questions like if Women experience more Discrimination and Stigma associated with Depression in comparison to the Depressive Men patients in the patriarchal culture of Pakistan? Study of Stigmatization in developing countries is very essential. This can serve as a baseline, as well as a first step in developing awareness in general public and the professionals about mental illnesses.

## METHOD

### Sample

The data used in this study is derived from an International Multisite Research^14^ which aimed to study the global pattern of Experienced and Anticipated Discrimination reported by people with Major Depressive Disorder. This Cross Sectional study has following Inclusion Criterias: Clinical diagnosis of Major Depressive Disorder (single episode or recurrent) diagnosed according to the Diagnostic and Statistical Manual of Mental Disorders (Fourth Edition, Text Revision) criteria in past 12 months. Patients with age range of 18 years and above. The participants have the ability to understand and speak Urdu Language.

### Exclusion Criteria

Those individuals who had been given psychotherapy as inpatient at some point in recruitment were excluded.

### Tools

### Discrimination & Stigma Scale

DISC^15^ contains 32 questions regarding different life aspects, which include marriage, work, housing, parenting, religious and leisure activities. Responses are rated on 4 point Likert Scale. The tool was translated into Urdu language for this study. The Internal Consistency of the scale for the present sample was 0.67 that is reliable.

### Internalized Stigma of Mental Illness Inventory

ISMI^16^ contains 29 items about Feeling Alienation, Stereotype Endorsement, Perceived Discrimination, Social Withdrawal, and Stigma Resistance. For the current study, tool was translated in Urdu language. The test re-test reliability of ISMI is 0.92. The Cronbach alpha value of the scale is reasonably good i.e. 0.82. However the Internal Consistency of ISMI subscales are; Alienation.64, Stereotype Endorsement is .63, and for Social Withdrawal it is 0.69.

Statistical Analyses was done using IBM SPSS Statistics (SPSS 21.0, Chicago, Illinois: SPSS Inc., 2015).

## RESULTS

The total number of participants were 38 patients diagnosed with Major Depressive Disorder including 21 Women and 17 Men, within age range of 20-65 years (M = 35.86, SD = 12.20). Sixty one percent were married and 53 % were living with their spouses and/or children. Majority of participants (61 %) were unemployed. Sixteen participants had more than six episodes of Depression (Table-I).

**Table-I:** Showing demographic characteristics of sample (N = 38).

Variables	f	%
*Gender*		
Men Participant	17	45
Women Participants	21	55
*Marital Status*		
Married	23	61
Separated, Widowed, Divorced	14	36
Single	1	3
*Living Status*		
Alone	5	13
With Spouse and/or Children	20	53
With Other Relatives, Unrelated People, Homeless	13	34
*Work Status*		
Full Time Job	13	34
Part Time Job	2	5
Unemployed	23	61
More than 6 Life Time Episodes of Depression	16	42

The mean level of Discrimination among Men was 73 (SD = 21.61) and the mean score for Women was 88 (SD = 31.45); t(34) = 1.55, p = .66, two tailed, 95% CI [33.66, 4.52] which indicate Non-Significant Mean Difference between these two groups. However on Stigma Scale, Women participants experienced Internalized Stigma (M = 76.32, SD = 16.04) more than Men participants (M = 67.71, SD = 10.15). *t*(34) = -1.94, *p = .04* which indicate a Significant Mean Difference between the two groups. However, it represent a medium-sized effect *r* = 0.31. Table-II.

**Table-II:** Independent sample t-test for mean differences on discrimination and internalized stigma associated with depression experienced by men and women.

Variables	Men		Women				95% CI	
	M	SD	M	SD	t	p	LL	UL
Discrimination	73.88	21.61	88.44	31.45	1.55	0.66	33.66	4.52
Internalized Stigma	67.71	10.15	76.32	16.04	-1.94	0.04	17.64	0.42
ISMI Alienation	13	2.85	15.84	4.23	-2.33	0.09	5.31	0.36
ISMI Stereotype Endorsement	16	3.14	18.37	5.15	-1.64	0.06	5.30	0.57
ISMI Discrimination Experience	11.71	2.02	13.42	4.22	-1.58	0.01	3.95	0.51
ISMI Social Withdrawal	13.82	3.12	15.84	4.47	-1.58	0.04	4.62	0.58
ISMI Stigma Resistance	13.18	2.09	12.84	2.19	0.46	0.80	1.12	1.79

ISMI = Internalized Stigma of Mental Illness; CI=Confidence Interval; LL=Lower Limit; UL=Upper Limit.

Subsequently on Subscales of ISMI, Women experienced more Internalized Stigma compared to Men. The significant difference was found among the two groups on subscales of Discrimination Experience *t*(34) = -1.58, *p = 0.00* (Men: M = 11.71, SD = 2.02; Women: M = 13.42, SE = 4.22) and Social Withdrawal *t*(34) = -1.58, *p = .04* (Men: M = 13.82, SE = 3.12) (Women: M = 15.84, SE = 4.47).

## DISCUSSION

The results from this study have shown that Discrimination and Stigma is experienced by both Men and Women; however Stigma is more experienced by Women who were diagnosed with Depression. The mean score of Experienced and Anticipated Discrimination measured with Discrimination and Stigma Scale (DISC) was high in Women in comparison to Men but not to the level of statistical significance. The mean score of Internalized Stigma measured with Internalized Stigma of Mental Illness Inventory (ISMI) was high in Women in comparison to Men at significance level of .05. Among the five subscales of Internalized Stigma; the significant difference was found on Discrimination Experience and Social Withdrawal.

The fear or anticipation of being stigmatized by the society cause avoidance in participation in particular life areas; hence lead individual towards segregation and social banishment.^17^ These results were replicated in previous studies, a study reported association between Anticipated and Experienced Discrimination, in which 87% of participants experienced Discrimination in at least one area of life in their past year.^17^,^18^ Moreover, Women anticipate and experienced more Discrimination than Men.^17^ A Qualitative Analysis of INDIGO Study on patients diagnosed with Schizophrenia; highlighted the negative feelings experienced by the mentally ill individuals in their communities; resulting in Social Withdrawal.^19^ Hence these Discrimination experience resulted in high prevalence of negative experiences leading individuals towards social banishment. 72% experienced Discrimination because of their mental illness; 50% reported that the negative stereotype and feeling of burdening others cause Social Withdrawal in them; 37% reported that they avoid getting close to those who are not mentally ill, to avoid rejection.^20^ The same results were replicated by another study which highlight that Social Withdrawal is the reason of the Stigma associated with people having Mental Illness.^21^ The perception of being Stigmatized and being treated discriminately was related to both Gender and Race. Hence studies report reluctance in such individuals to seek help due to Perceived and Experienced Stigma.^16^

### +The plausible explanation for these findings could be following

The Patriarchal Culture of Pakistan provides more tolerability to Men’s problem in comparison to the Women. This societal trend engulfs all aspect of life like; illness, health, employments, responsibilities and decision making. Secondly, generally Women have greater level of psychological distress in comparison to the Men. So they are prone to experience greater level of Discrimination and Stigma then Men.^22^ The present study is first of its kind that has examined the gender differences between the Discrimination and Internalized Stigma experienced by Depressed Patients in Pakistan. It suggested that Internalized Stigma (Stereotypical Endorsement, Perceived Discrimination, Social Withdrawal, Feeling Alienated and Stigma Resistance) should target specifically treatment of Depression. Consequently Stigma and Discrimination affects Life Quality, Self-Esteem and Sense of Well Being.^6^ The current study is kind of first evidence that to what extent people suffering with Depression are also carrying the burden of Stigma and Discrimination in Pakistan. This study reveals actual experiences of Discrimination and Stigma are ascertained rather than hypothetical scenarios.

Further researches can unveil many solutions in better coping with Stigma and Discrimination linked with Mental Illnesses and in generating Health Policies at National Level.

Nevertheless, the study has certain Limitations. Selected participants were representing only those patients who were sorting treatment and not the prevalent cases thus the generalizability of this study is compromised. Causal relationship between possible predictors and discrimination cannot be ascertained due to the Cross Sectional Research Design.

The findings suggest that efforts are required to be made at multiple levels in third world country like Pakistan. Media campaigns can increase the awareness of Mental Illness and consequently reduce the Public Stigma and Internalized Stigma and Perceived Discrimination among patients with psychological problems.

Efforts should also be made at Individual, Institutional and Societal Level to minimize the effect of Discrimination and Stigma experienced by people with Depression. Social participation of client should be enhanced to reduce or eliminate Internalized Stigma. It is also suggested in the light of this study that Women need greater attention for dealing with Discrimination and Stigma.

It is concluded that Discrimination and Internalized Stigma is affecting different life domains of the patients diagnosed with Depression so sustainable and affective approaches are required to prevent, reduce and eliminate the Discrimination and Stigma faced by people with Mental Illness.
